# Motilin Stimulates Gastric Acid Secretion in Coordination with Ghrelin in *Suncus murinus*

**DOI:** 10.1371/journal.pone.0131554

**Published:** 2015-06-26

**Authors:** Chayon Goswami, Yoshiaki Shimada, Makoto Yoshimura, Anupom Mondal, Sen-ichi Oda, Toru Tanaka, Takafumi Sakai, Ichiro Sakata

**Affiliations:** 1 Area of Regulatory Biology, Division of Life Science, Graduate School of Science and Engineering, Saitama University, Saitama, Japan; 2 Laboratory of Animal Management and Resources, Department of Zoology, Okayama University of Science, Okayama, Japan; 3 Faculty of Pharmaceutical Sciences, Josai University, Saitama, Japan; Medical University of Gdańsk, POLAND

## Abstract

Motilin and ghrelin constitute a peptide family, and these hormones are important for the regulation of gastrointestinal motility. In this study, we examined the effect of motilin and ghrelin on gastric acid secretion in anesthetized suncus (house musk shrew, *Suncus murinus*), a ghrelin- and motilin-producing mammal. We first established a gastric lumen-perfusion system in the suncus and confirmed that intravenous (i.v.) administration of histamine (1 mg/kg body weight) stimulated acid secretion. Motilin (0.1, 1.0, and 10 μg/kg BW) stimulated the acid output in a dose-dependent manner in suncus, whereas ghrelin (0.1, 1.0, and 10 μg/kg BW) alone did not induce acid output. Furthermore, in comparison with the vehicle administration, the co-administration of low-dose (1 μg/kg BW) motilin and ghrelin significantly stimulated gastric acid secretion, whereas either motilin (1 μg/kg BW) or ghrelin (1 μg/kg BW) alone did not significantly induce gastric acid secretion. This indicates an additive role of ghrelin in motilin-induced gastric acid secretion. We then investigated the pathways of motilin/motilin and ghrelin-stimulated acid secretion using receptor antagonists. Treatment with YM 022 (a CCK-B receptor antagonist) and atropine (a muscarinic acetylcholine receptor antagonist) had no effect on motilin or motilin-ghrelin co-administration-induced acid output. In contrast, famotidine (a histamine H_2_ receptor antagonist) completely inhibited motilin-stimulated acid secretion and co-administration of motilin and ghrelin induced gastric acid output. This is the first report demonstrating that motilin stimulates gastric secretion in mammals. Our results also suggest that motilin and co-administration of motilin and ghrelin stimulate gastric acid secretion via the histamine-mediated pathway in suncus.

## Introduction

It is well established that gastric acid secretion is regulated by neurocrine, endocrine, and paracrine signals [1]. Histamine, acetylcholine, and gastrin are the major direct peripheral stimuli acting on the parietal cells for gastric acid secretion [1], and histamine is the most effective stimulating factor to these cells. Recently, several reports have demonstrated that ghrelin and other gut hormones also stimulate gastric acid secretion through histamine mediated pathways [2, 3].

Ghrelin was originally isolated from rat and human stomachs [4] and its central or peripheral administration causes the initiation of food intake in many species [5–9]. In addition, it is well known that ghrelin is a multifunctional hormone; many studies revealed that ghrelin is important for the regulation of gastrointestinal tract [4, 6, 10–14], in particular, it stimulates gastric acid secretion in the rat [15], and also induces gastric motility by peripheral or central administration [16, 17]. Motilin, produced mainly in the duodenum, is considered to be of the same peptide family as ghrelin [18, 19]. It has been well documented that gastric contraction is strongly associated with plasma motilin levels and that the intravenous administration of motilin induces gastric phase III-like contractions in humans [20, 21] and dogs [22–24]. Recently, we reported that motilin and ghrelin synergistically stimulated gastric contraction in suncus (house musk shrew, *Suncus murinus*), suggesting that focusing on the coordination of motilin and ghrelin is important for understanding their physiological role [25]. Previous studies have shown that cyclic release of motilin in an interdigestive period induces phase III contraction of the migrating motor complex (MMC) in the stomach. However, the causes and mechanism for the ending of phase III contractions are not clearly understood. We hypothesized that the peaked concentration of motilin may be involved in the extinction of phase III contractions, and thus a return to the basal level of phase I contraction. Studies have also shown that interdigestive acid secretion in the stomach disrupts the regular occurrence of phase III contractions [26, 27]. Therefore, we studied the effect of motilin on gastric acid secretion.

Although motilin was discovered decades ago, there have been limited studies on the physiological functions of motilin, other than gastrointestinal motility, because the study of the biological action of motilin beset with several problems. The greatest difficulty in study of motilin is the lack of a suitable small laboratory animal for experimentation. Since rodents lack the genes for motilin and its receptors [28], these commonly used experimental animals cannot be employed for studies on motilin. Recently, we focused on the suncus as a laboratory animal for gastrointestinal studies using motilin-ghrelin family peptides. Under the fasting condition, the repeated occurrence of MMCs was observed in suncus and motilin stimulated gastric contraction in a dose-dependent manner in humans and dogs alike [29, 30]. In addition, complete cDNA sequences and the tissue distribution of motilin and ghrelin were identified in suncus [30, 31]. These results suggest that suncus is a useful animal for studying gastrointestinal physiology, including gastric motility.

In the present study, we established an experimental procedure for acid secretion using the stomach perfusion system in anesthetized suncus, and studied the effect of ghrelin and motilin on acid secretion. In addition, we also examined the acid secretion mechanism by motilin and co-administration of motilin and ghrelin, using receptor antagonists.

## Materials and Methods

### Animals

Experiments were performed using adult male (10–30 weeks of age) and female (5–30 weeks of age) suncus of an outbred KAT strain established from a wild population in Kathmandu, Nepal [32], weighing between 50 and 100 g. Animals were housed individually in plastic cages equipped with an empty can for a nest box under controlled conditions (23 ± 2°C, lights on from 8:00 to 20:00) with free access to water and commercial feeding pellets (number 5P; Nippon Formula Feed Manufacturing, Yokohama, Japan). The metabolizable energy content of the pellets was 344 kcal/100 g. The pellets consisted of 54.1% protein, 30.1% carbohydrates, and 15.8% fat. All procedures were approved and performed in accordance with the Committee on Animal Research of Saitama University (Saitama, Japan). All efforts were made to minimize animal suffering and to reduce the number of animals used in the experiment.

### Drugs

Gastric acid secretion was stimulated by intravenous (i.v.) bolus infusion of histamine dihydrochloride (Nakarai Chemicals Co., Ltd., Kyoto, Japan), pentagastrin (Sigma Aldrich, USA), carbachol (Tocris Bioscience, Ellisville, MO), suncus motilin (Scrum Inc., Tokyo, Japan), and human acylated ghrelin (Asubio Pharma Co., Ltd., Hyogo, Japan). Famotidine (Wako Pure Chemical Industries Ltd.), YM 022 (Sigma-Aldrich), and atropine sulfate (Mylan Pharma, Osaka, Japan) were used as H_2_ receptor, CCK-B receptor, and muscarinic cholinergic receptor antagonist, respectively. Motilin, ghrelin, and pentagastrin were dissolved in 0.1% BSA in PBS (phosphate-buffered saline), while histamine, carbachol, and atropine were dissolved in 0.9% saline. Famotidine was dissolved in 0.5 N HCl, and i.v. administration was performed after dilution in 0.1% BSA in PBS. YM 022 was dissolved in dimethylsulfoxide (DMSO) and subsequent dilutions were made in saline containing DMSO. All solutions were prepared immediately before each experiment. The dosage of each agent was administered at a rate of 100 μL per 100 g BW.

### Determination of gastric acid output by an intragastric perfusion experimental system

Overnight-fasted animals were anesthetized with an intraperitoneal injection of 15% urethane solution at a dose of 1 ml/100 g body weight, and prepared for the gastric acid output study by using intragastric perfusion system (S1 Fig). After anesthesia, the animals' tracheas were exposed, cannulated, and exteriorized to avoid choking due to catheter insertion through esophagus. Then, a catheter (polyethylene tube) was inserted into the stomach from the mouth and the tip of the catheter was placed about 3 mm from the cardia inside the stomach; the tube was fixed to the esophagus with a suture. Then, the abdomen was opened through the linea alba to expose the stomach. The pyloroduodenal junction was then exposed, and another polyethylene tube was introduced into the stomach via an incision in the duodenum, and was secured firmly with a ligature around the pylorus. Then, we exposed the jugular vein to insert a cannula for administration of reagents/drugs. The stomach lumen was washed with saline until the effluent was clear and then perfused with saline solution at 37°C at a rate of 0.25 ml/min using a peristaltic pump (Micro tube pump MP-3EYELA; Tokyo Rikakikai Co., Ltd., Tokyo). The stomach was perfused with saline through the liquid discharge tube and gastric output was collected through the perfusion catheter. In order to stabilize the amount of acid secretion, the pH of the collected solution was allowed to stabilize for 60 minutes from the start of the perfusion of saline. Effluents were collected in the tube continuously at 10-min intervals by a DF-2000 fraction collector (Tokyo Rikakikai Co., Ltd., Tokyo, JAPAN). We measured the pH of the gastric output with a pH meter (HORIBA Scientific, LAQUA ELECTRODE, HORIBA Ltd., JAPAN). We determined the acid output by a neutralization titration using 0.01 N NaOH solution. The amount of acid secretion was expressed per 10 minutes as H^+^ μEq. The amount of change in gastric secretion was measured by deducting the area under the curve (AUC) of the gastric acid secretion at 50 min before and after the administration of each drug. The values are expressed as ΔμEq/50 min.

### Experimental protocols

Intravenous (i.v.) bolus infusion of histamine dihydrochloride at the dose of 1 mg/kg BW [33], human acylated ghrelin (0.1, 1, and 10 μg/kg BW), and suncus motilin (0.1, 1, and 10 μg/kg BW) was done to study their stimulatory effect on acid secretion. A previous study confirmed that seven amino acids with acyl modification on the third Ser residue in the N-terminal region of ghrelin showed full biological activity [34]. In addition, the first 10 amino acids of mature suncus ghrelin sequence are identical among other mammals, including humans [31], indicating that human ghrelin is enough to exert full biological activity in suncus. Therefore, we used human ghrelin in this experiment. Combined effects of motilin and ghrelin were studied by the co-administration of motilin and ghrelin at doses of 0.1, 1, and 10 μg/kg BW (each). Based on the dose response, motilin and co-administration of motilin and ghrelin at 10 μg/kg BW were selected for all further studies. Famotidine (0.33 mg/kg BW) [35] was used to evaluate the role of the histamine (H_2_) receptor on motilin and co-administration of motilin and ghrelin stimulating gastric acid secretion. After confirming that pretreatment of famotidine (0.33 mg/kg BW) completely inhibited histamine-stimulated (1 mg/kg BW) acid secretion, famotidine at the pretreatment dose was administered 30 min before administration of motilin (10 μg/kg BW) and co-administration of motilin (10 μg/kg BW) and ghrelin (10 μg/kg BW). To examine the involvement of gastrin (CCK-B) receptors in motilin-stimulated gastric acid secretion, we also administered YM 022 (0.2 mg/kg BW) [36, 37]. Vehicle or YM 022 (0.2 mg/kg BW) was administered 30 min before each drug injection. Similarly, an mACh receptor antagonist, atropine (30 μg/kg BW) was also administered 30 min before motilin or motilin/ghrelin co-administration to examine the influence of these receptors on gastric acid secretion.

### Statistical analyses

We repeated the recording experiments individually at least three times. All data are indicated as mean ± S.E.M. We used GraphPad Prism 5 software (GraphPad Software Inc., CA, USA) to analyze the data. Statistical analyses were performed using a one-way analysis of variance (ANOVA) followed by Dunnett's Multiple Comparisons Test or Student’s *t* test. *p* < 0.05 was considered statistically significant.

## Results

### Effect of ghrelin, motilin, and co-administration of motilin and ghrelin on gastric acid secretion

**Fig 1:**
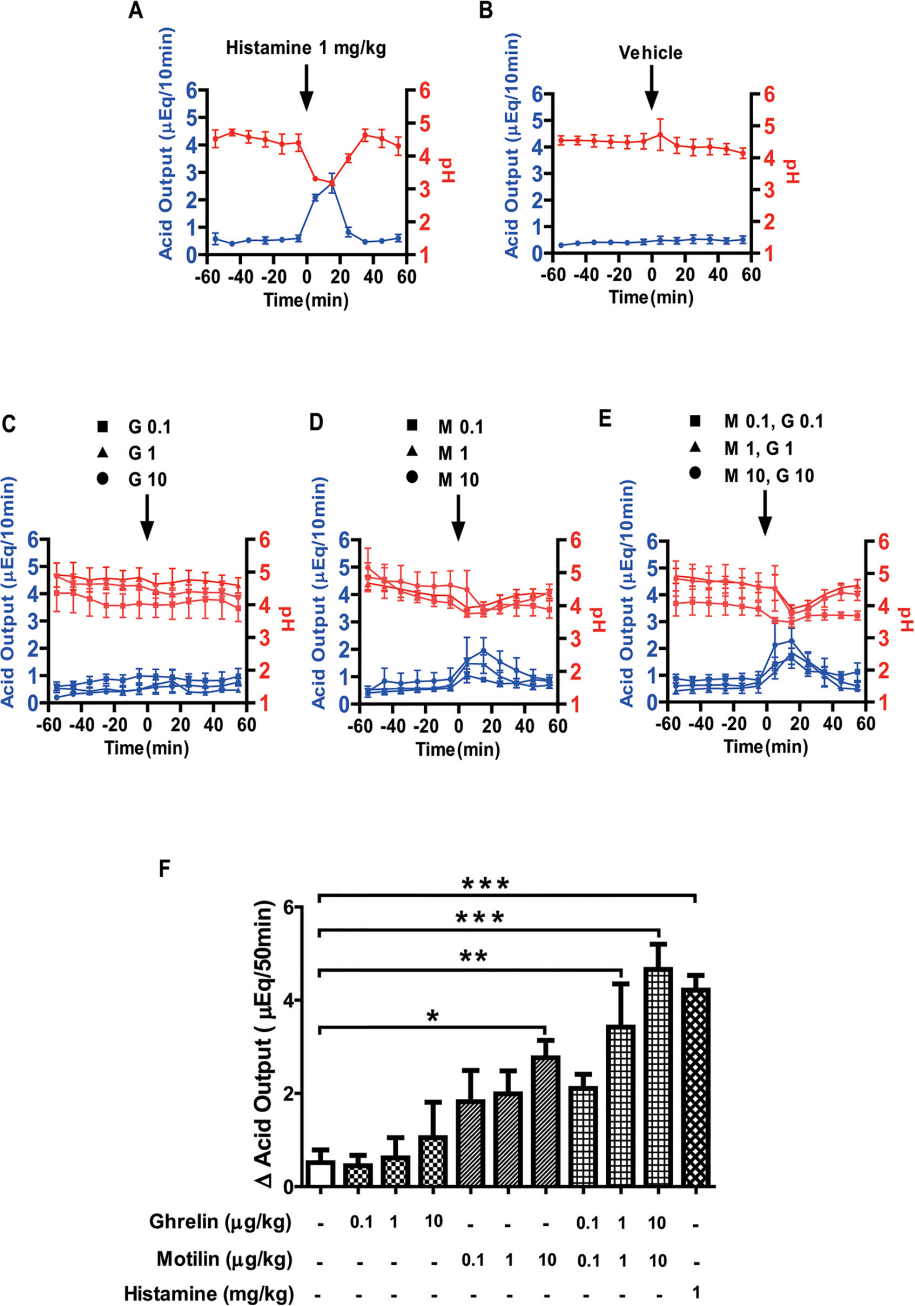
Effects of motilin and ghrelin on gastric acid secretion. In urethane-anaesthetized suncus, after a 60-min basal period, gastric acid secretion was measured after administration of histamine (1 mg/kg BW) (A), vehicle (B), ghrelin (0.1, 1.0, and 10 μg/kg BW) (C), motilin (0.1, 1.0, and 10 μg/kg BW) (D), and co-administration of motilin and ghrelin (E). Gastric acid secretion (blue line) and pH (red line) changes were monitored at 10-min intervals throughout the experiment. The net change in cumulative acid output for 50 min after each administration was also calculated (F). Each value represents the mean ± SEM. p < 0.05 was considered statistically significant. G: ghrelin; M: motilin, figures after the abbreviations denote concentration in μg/kg. n = 3–7.

To establish the perfusion system for gastric acid experiment in suncus, we first examined the effect of histamine (1 mg/kg) on acid secretion (Fig 1A). Intravenous (i.v.) administration of histamine started to increase the acid output after 10 min and reached its maximum at 20 min, after which the acid output reduced to baseline around 30–40 min after histamine administration (Fig 1A). The pH of the gastric output had maximally decreased 20 min after histamine administration and restored to its previous values at 40 min after administration (Fig 1A). The amount of acid secretion induced by 1 mg/kg histamine was 4.22 ± 0.32 μEq/50 min and the acid output significantly increased compared to the vehicle (control: 0.52 ± 0.27 μEq/50 min) (Fig 1F). Vehicle administration (0.1% BSA in PBS) did not change the basal gastric acid secretion (Fig 1B). The i.v. administration of ghrelin at the doses of 0.1, 1.0, and 10 μg/kg did not increase the acid output and the pH was slightly decreased, but not significantly so (Fig 1C). In contrast, i.v. administration of motilin started to increase acid secretion after 10 min and reached its maximum at 20 min after administration (Fig 1D). The motilin-induced acid secretion returned to baseline 40 min after administration and the pH value of the gastric output decreased within 30–40 min of motilin administration (Fig 1D). The amount of gastric acid secretion 50 min after motilin administration at the doses of 0.1, 1.0, and 10 μg/kg increased dose-dependently (Fig 1F). Co-administration of motilin and ghrelin (0.1, 1.0, and 10 μg/kg BW each) also induced the secretion of gastric acid and it reached its maximum in 20 min after administration, and decayed gradually before 50 min had elapsed (Fig 1E). A maximum decline of gastric pH was observed within 20 min and returned to baseline 50 min after the administration of each dose (Fig 1E). The amount of gastric acid secretion 50 min after co-administration of motilin and ghrelin increased in a dose-dependent manner (Fig 1F). Statistical analysis showed that 10 μg/kg motilin, and co-administration of motilin and ghrelin (1 and 10 μg/kg, each) significantly increased acid output compared to vehicle administration (Fig 1F).

### Effect of famotidine on motilin, and co-administration of motilin and ghrelin-stimulated gastric acid secretion

**Fig 2:**
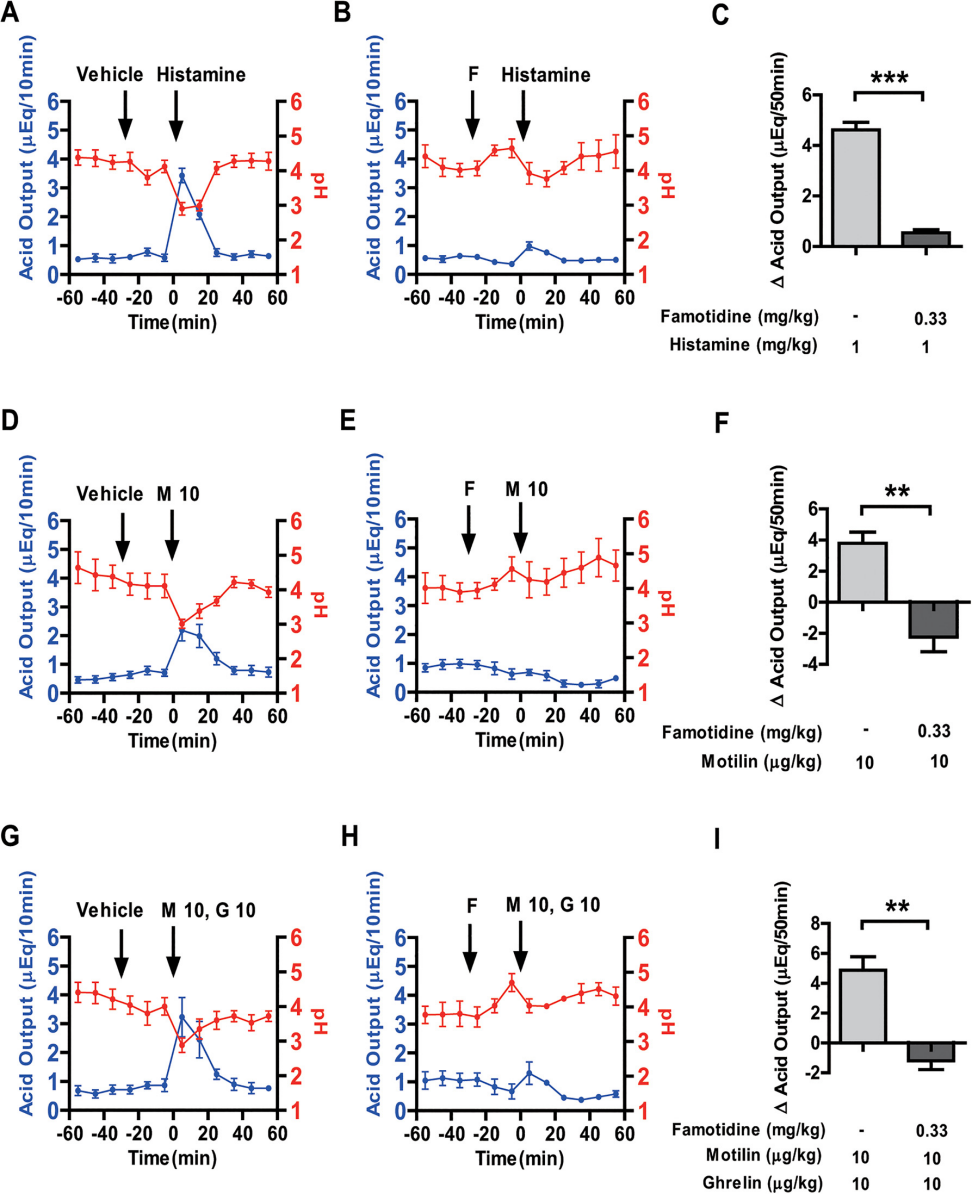
Effects of famotidine on motilin, and co-administration of motilin and ghrelin-stimulated gastric acid secretion. Gastric acid secretion was stimulated with histamine (1 mg/kg BW) (A, B), motilin (10 μg/kg BW) (D, E) and co-administration of motilin (10 μg/kg BW) and ghrelin (10 μg/kg BW) (G, H) with or without famotidine (0.33 mg/kg BW). In the left and middle panels, vehicle/famotidine was administrated intravenously 30 min before histamine (A, B), motilin (D, E) and co-administration of motilin and ghrelin (G, H). Gastric acid secretion (blue line) and pH (red line) changes were monitored at 10-min intervals throughout the experiment. Right panels represent the net change in cumulative acid output for 50 min after administration of histamine (C), motilin (F) and co-administration of motilin and ghrelin (I) with pre-administration of vehicle or famotidine. Each value represents the mean ± SEM. *p* < 0.05 was considered statistically significant. F: famotidine; G: ghrelin; M: motilin, figures after the abbreviations denote concentration in μg/kg. n = 3–4.

We next examined the effect of famotidine (an H_2_ receptor antagonist) on motilin, and co-administration of motilin and ghrelin-stimulated gastric acid secretion. Famotidine (0.33 mg/kg) significantly decreased histamine-induced acid output and also inhibited the decrease in pH by histamine treatment (Fig 2A–2C). In order to examine whether the H_2_ receptor is the mediator of motilin and combined motilin and ghrelin-stimulated acid secretion, i.v. administration of vehicle and famotidine (0.33 mg/kg) was done 30 min before the administration of motilin (10 μg/kg) and co-administration of motilin (10 μg/kg) and ghrelin (10 μg/kg). Famotidine treatment gradually decreased baseline acid secretion; however, no peak was observed after motilin administration. The stimulatory effect of motilin on gastric acid secretion was completely inhibited by famotidine treatment (Fig 2D–2F). Similarly, the effect of co-administration of motilin and ghrelin was almost completely eliminated by famotidine treatment (Fig 2G–2I).

### Effect of YM 022 on motilin, and co-administration of motilin and ghrelin-stimulated gastric acid secretion

**Fig 3:**
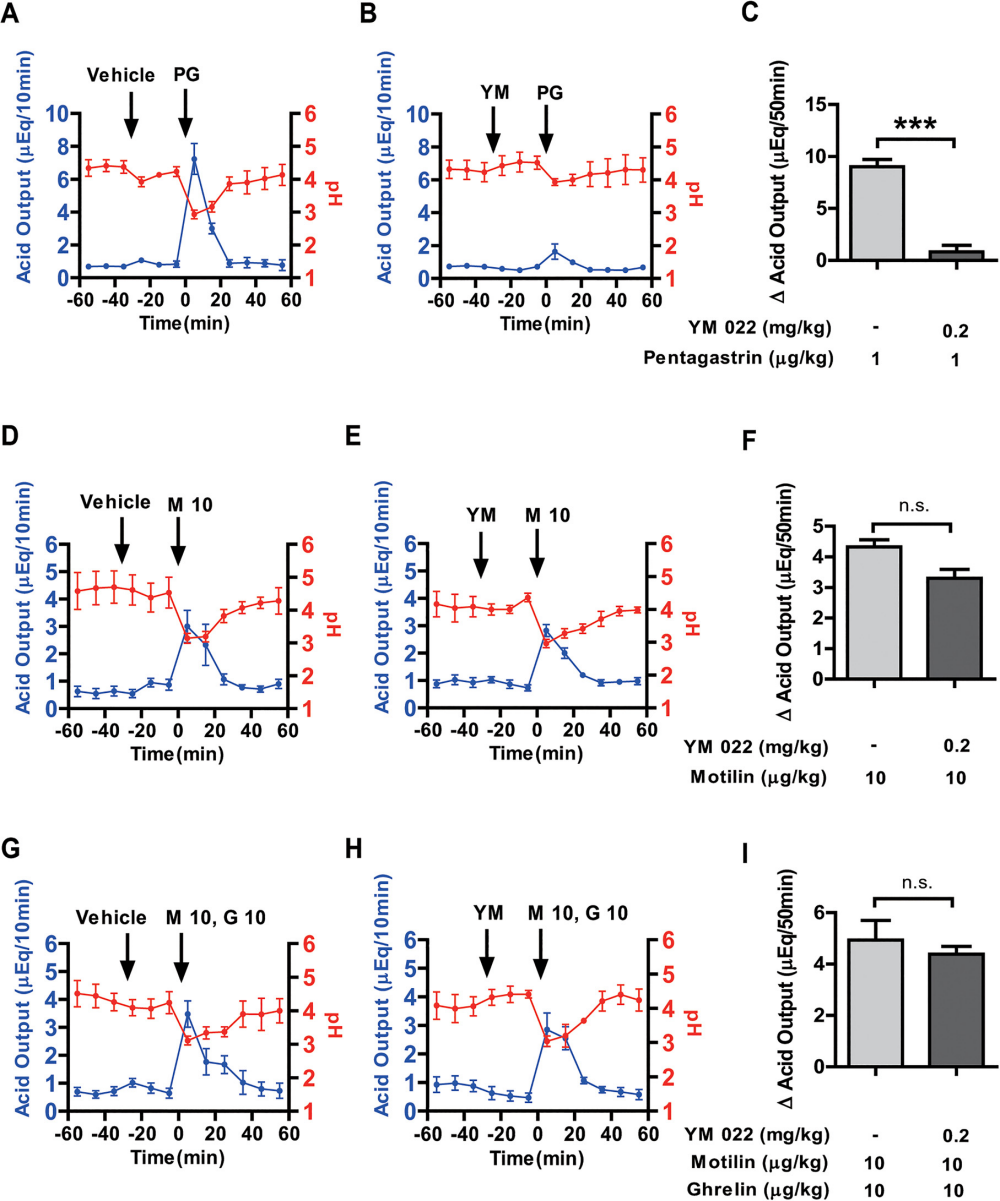
Effects of YM 022 on gastric acid secretion stimulated by motilin, and co-administration of motilin and ghrelin. Left and middle panels represent the gastric acid secretion (blue line) and pH (red line) changes at 10-min intervals after intravenous administration of vehicle/YM 022 (0.2 mg/kg BW) followed by pentagastrin (1 μg/kg BW) (A, B), motilin (10 μg/kg BW) (D, E) and co-administration of motilin and ghrelin (G,H) throughout the experiment. The net change in cumulative acid output for 50 min after administration of pentagastrin (C), motilin (F) and co-administration of motilin and ghrelin (I) with or without YM 022 pretreatment is shown in the right panels. Each value represents the mean ± SEM. *p* < 0.05 was considered statistically significant. YM: YM 022; PG: pentagastrin; G: ghrelin; M: motilin, figures after the abbreviations denote concentration in μg/kg. n = 3.

To examine the involvement of gastrin mediated pathways for motilin-stimulated gastric acid secretion, we used YM 022 (a CCK-B receptor antagonist). Pentagastrin (1 μg/kg) stimulated acid secretion (8.95 ± 0.73 μEq/50 min) continued for 30 min, but this increase was significantly and almost completely attenuated (0.82 ± 0.62 μEq/50 min) by pretreatment with YM 022 (0.2 mg/kg) (Fig 3A–3C). In contrast, gastric acid secretion stimulated by the motilin treatment did not change with pretreatment of YM 022 (Fig 3D and 3E). The stimulatory effect of motilin (10 μg/kg BW) on gastric acid secretion after vehicle/YM 022 pre-administration was statistically insignificant over 50 min (Fig 3F). Furthermore, we also checked the effect of YM 022 on gastric acid secretion stimulated by motilin and ghrelin co-administration. However, no effect was found for YM 022 pretreatment (Fig 3G–3I).

### Effect of atropine on gastric acid secretion stimulated by motilin, and co-administration of motilin and ghrelin

**Fig 4:**
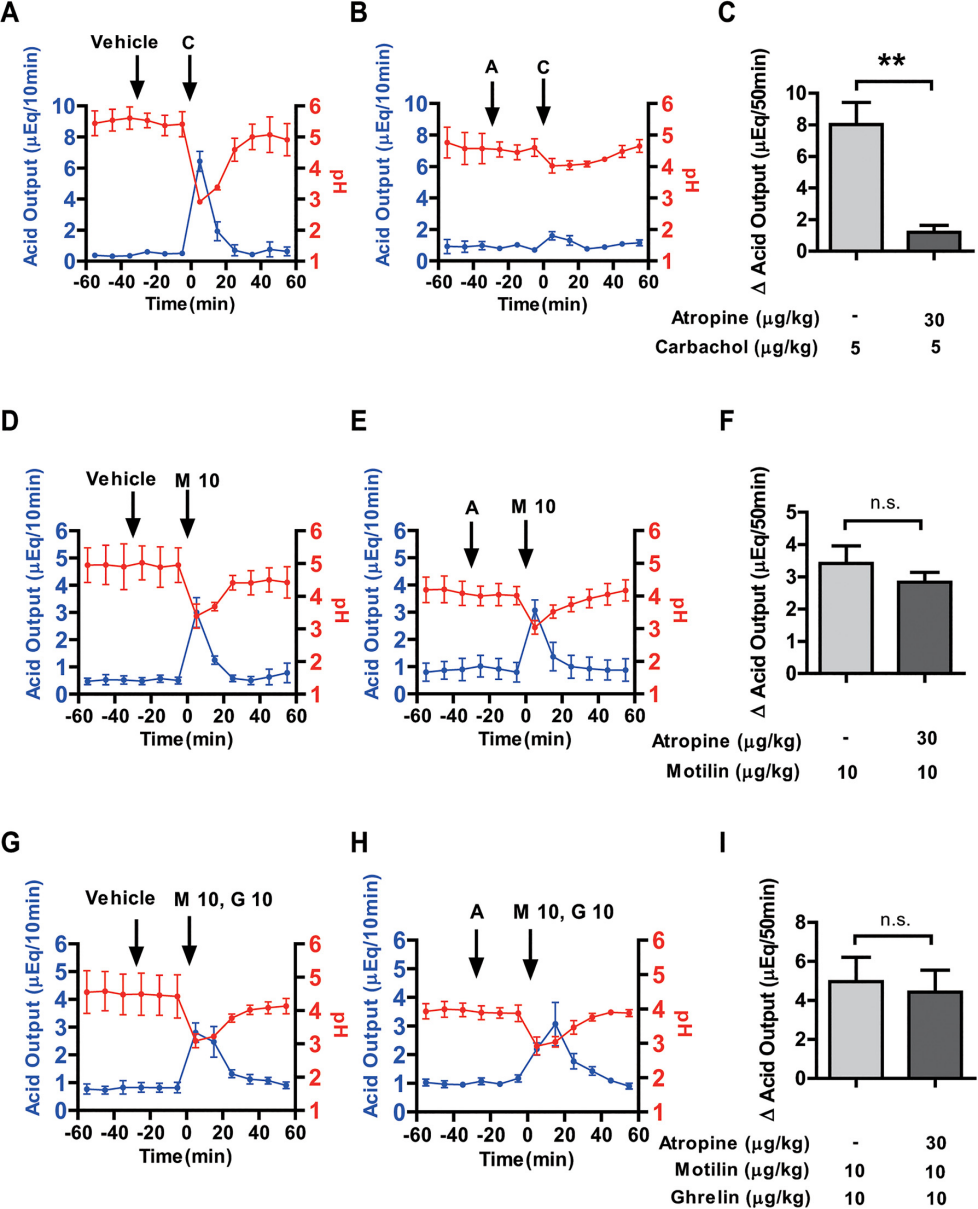
Effects of atropine on motilin, and co-administration of motilin and ghrelin-stimulated gastric acid secretion. Gastric acid secretion (blue line) and pH (red line) changes were monitored at 10-min intervals throughout the experiment. In the left and middle panels, intravenous administration of either vehicle or atropine (30 μg/kg BW) was done 30 min before carbachol (5 μg/kg BW) (A, B), motilin (10 μg/kg BW) (D, E) and co-administration of motilin and ghrelin (G,H) treatment. Right panels compare the net change in cumulative acid output for 50 min after administration of carbachol (C), motilin (F) and co-administration of motilin and ghrelin (I) with vehicle or atropine pretreatment. Each value represents the mean ± SEM. *p* < 0.05 was considered statistically significant. A: atropine; C: carbachol; G: ghrelin; M: motilin, figures after the abbreviations denote concentration in μg/kg. n = 3.

Thirty minutes of pre-administration with atropine (30 μg/kg BW) almost completely inhibited the carbachol (5 μg/kg BW) stimulated acid secretion (Fig 4A–4C). Vehicle pretreatment had no effect on motilin (10 μg/kg BW) stimulated gastric acid secretion (Fig 4D). However, pretreatment with atropine could not inhibit the motilin-stimulated gastric acid output (Fig 4E). Furthermore, net change in cumulative gastric acid output by motilin for 50 min was not changed by atropine pretreatment (Fig 4F). Similarly, atropine pretreatment before motilin and ghrelin co-administration did not suppress gastric acid secretion (Fig 4G–4I).

## Discussion

We used suncus as a model animal to study the effect of motilin and/or ghrelin on gastric acid secretion. Suncus, which belongs to the order Insectivora, was first established as a model animal for research on emesis and vomiting [38, 39]. Recently, we found that this animal produced not only motilin but also its receptor [40]. Considering that the motilin gene in rodents, the most widely used small laboratory animal, is converted into a pseudogene and lacks gastric contraction in response to motilin treatment [18, 28], suncus might be a unique small animal model for studies on motilin [30]. The mucosa of suncus is similar to that of humans and dogs [41], and also has an almost identical MMC of gastrointestinal motility, and motilin responses, as that found in humans and dogs [29]. In addition, the coordinating effect of motilin and ghrelin was also observed in regulating the MMC of gastrointestinal motility in suncus [25]. In this study, we focused on the effect of motilin on gastric acid secretion by using suncus.

In previous studies, it has been demonstrated that intravenous administration of ghrelin stimulates acid secretion in a dose-dependent manner in the rat through vagal afferent nerve [15–17]. However, in this study, intravenous administration of ghrelin showed no significant increase in acid secretion even at high concentrations (10 μg/kg). On the basis of our results that motilin stimulated gastric acid output in a dose-dependent manner, ghrelin might act to compensate for the function of motilin in rodents because a similar example is found in rat gastric motility [17]. It has been reported that ghrelin is a strong prokinetic hormone in rats and mice [17, 42]. However, in the suncus and humans, ghrelin has less potent effects on gastric contraction compared to that of motilin [21, 25, 29], suggesting that ghrelin stimulates gastric contraction instead of motilin. It is interesting to study the effect of motilin and/or ghrelin on gastric acid output in motilin-producing animals such as humans and dogs.

We first found a novel physiological function for motilin as a stimulator of gastric acid secretion in suncus. In previous studies, plasma motilin concentration was found to increase at the start of gastric phase II in fasted dogs, and gradually reached a peak just after the termination of phase III in an interdigestive state [24, 43]; further, these motilin peaks were found at an interval of about 100 min, and induced phase III contractions of the MMC in human [21] and in dogs [24]. Moreover, Gielkens *et al*. reported that, in humans, the amount of gastric acid output during phase I and early phase II was low, but increased significantly during late phase II and reached its highest point during phase III of the MMC [44]. Taken together with our present results, this suggests that peak concentration of motilin may not only stimulate the phase III contraction but also gastric acid secretion. Further, many studies have clearly shown that stomach acidification attenuated spontaneous phase III and motilin-induced contractions. For example, Yamamoto *et al*. reported that antral acidification inhibits motilin-induced phase III contractions in the stomach of dogs through vagovagal reflux [45], and, similarly, intragastric acidification also inhibits the effect of motilin on antroduodenal motility and causes a delay in the occurrence of phase III in humans [27]. Furthermore, pentagastrin independently inhibits motilin-induced phase III-like contraction by gastric acidification via a CCK receptor-dependent mechanism [46]. Additionally, studies have also shown that motilin can induce gastric contraction after inhibition of gastric acid secretion by famotidine (an H_2_ receptor antagonist) in a patient with duodenal ulcers [47]. According to these studies, motilin-induced phase III contractions are suppressed by motilin-induced gastric acidification, and this may stop the phase III period even at high concentrations of motilin.

**Fig 5:**
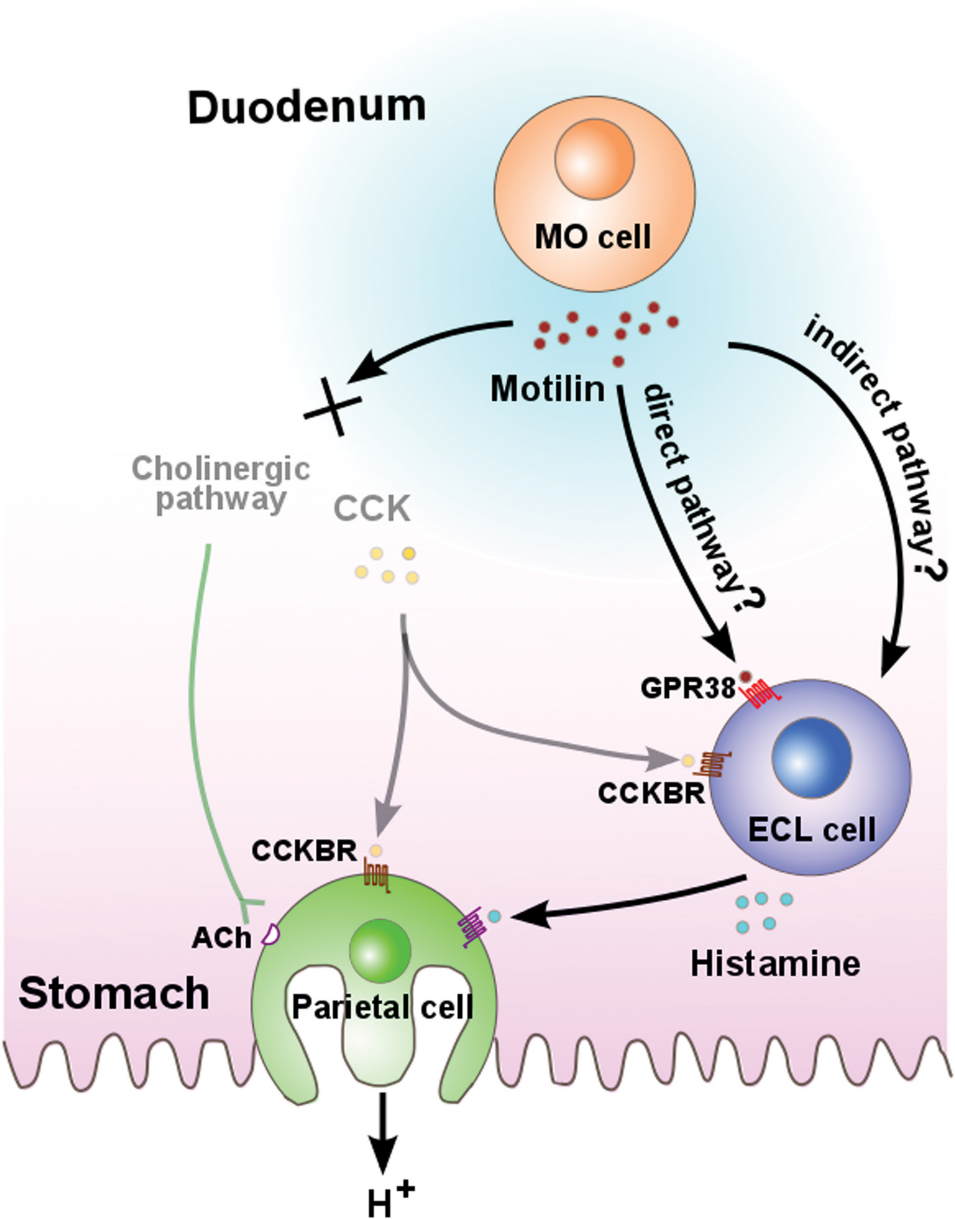
Working hypothesis of stimulation of acid secretion by motilin in suncus. In the duodenum, motilin is secreted by the MO cell during the interdigestive state. It was hypothesized that motilin may directly stimulate histamine release through GPR38 expressed on the ECL cell. Another possibility is the indirect pathway, which is either central or peripheral neural stimulation of motilin for histamine release. Finally, the histamine induced by motilin acts on the parietal cell through the H_2_ receptor to release gastric acid. CCKB and muscarinic cholinergic receptors were not found to be involved in motilin-induced gastric acid secretion. MO cell: motilin-producing cell; GPR38: G-protein coupled receptor 38; CCK: cholecystokinin; CCKBR: cholecystokinin B receptor; ECL cell: enterochromaffin-like cell; ACh: Acetylcholine.

In this study, the effect of the co-administration of low-dose (1 μg/kg) motilin and ghrelin, but not motilin (1 μg/kg) or ghrelin (1 μg/kg) alone, was found to significantly stimulate gastric acid secretion compared with the vehicle and was almost comparable with that of 10 μg/kg motilin alone. This suggests that the additive role of ghrelin in motilin-induced gastric acid secretion is close to the physiological concentration. Moreover, further studies are required to elucidate the detailed mechanism of ghrelin, especially the target site of ghrelin. Meanwhile, we demonstrated the mechanism of action of motilin on gastric acid secretion. As summarized in Fig 5, we observed that gastric acid secretion by motilin administration was completely eliminated by pretreatment with famotidine (an H_2_ receptor antagonist), but not atropine or gastric/CCK-B receptor antagonists, indicating that motilin-stimulated acid secretion was finally mediated by the H_2_ receptor in the stomach. In addition, the suncus genome database confirmed that the predicted mRNA sequence of the H_2_ receptor, which is highly conserved in mammals, shows high homology with that in humans (data not shown). This suggests that histamine and famotidine are a specific agonist and antagonist, respectively, for H_2_ receptor in suncus. Hormone stimulating gastric acid secretion through histaminergic neural pathway is not a rare occurrence; for example, famotidine can inhibit ghrelin-stimulated gastric acid secretion in the rat [2] and PACAP (pituitary adenylate cyclase activating polypeptide) has also induced histamine release via a PACAP type 1 receptor on enterochromaffin-like (ECL) cells [3]. Motilin also stimulates gastric acid secretion via a histaminergic pathway, as was found in the case of ghrelin and PACAP. However, details regarding the specific target site of motilin for histamine release is unknown to date. There are three possible pathways that can be considered. One candidate is the central pathway. This possibility is supported by our findings that GPR38 mRNA is expressed in the nodose ganglion and the medulla oblongata [40], suggesting motilin affects vagus afferents and may stimulate acid output at its cephalic phase. Peripheral neural stimulation is the second candidate, as we showed that motilin excited cholinergic neural pathways in the myenteric plexus in an *in vitro* study of suncus; this enteric neural pathway may affect ECL cells. As a third candidate, the direct action of motilin on ECL cells cannot be ruled out. Further studies are needed for complete understanding of the target site of motilin, as well as the precise mechanism of motilin-induced gastric acid secretion.

In summary, the present study provides new findings that ghrelin alone has no effect on gastric acid secretion but that motilin stimulates gastric acid secretion in a dose-dependent manner in suncus. Further studies using different animals, including humans, are needed to clarify the effect of motilin on gastric acid secretion. This may lead to useful knowledge in the clinical realm.

## Supporting Information

S1 Fig Schematic representation of perfusion setup for gastric acid secretion experiment in suncus. After anesthesia, a suncus was placed on a culture flask containing circulating hot water from a water bath. A logger was used to monitor body temperature during the experiment. Perfusion of saline solution was done by a peristaltic pump, and gastric output was collected through a catheter from the stomach at intervals of 10 minutes. Effluents were used to measure pH and acid output. Intravenous drug administration was done through the jugular vein.
(EPS)
